# Emerging trends in genome editing of wild animals

**DOI:** 10.1007/s11248-026-00483-y

**Published:** 2026-02-06

**Authors:** Torill Blix, Anne Ingeborg Myhr

**Affiliations:** NORCE Research AS, Climate & Environment, Sykehusveien 21, 9019 Tromsø, Norway

**Keywords:** Conservation biotechnology, CRISPR, Biodiversity, Facilitated adaptation, Population control, De-extinction, GMO regulation

## Abstract

Globally, nearly one million species are currently threatened with extinction, highlighting the need for more efficient solutions to biological conservation. Genome editing, which allows for faster and more precise changes in genomes, is a promising technique for boosting populations through facilitated adaptation, management of invasive or pathogenic populations, and potentially even facilitating the revival of extinct species. These approaches belong to a new field of research termed conservation biotechnology, which places a great responsibility on researchers and decision makers to ensure sustainability. In this paper, we have mapped the emerging trends in genome editing of wild animals. Current projects primarily focus on population control and de-extinction, with fewer initiatives aimed at preserving threatened species. We then explore four critical dimensions of conservation biotechnology: the technology itself, new perspectives on conservation practices, research organization, and governance and policy. Despite its potential, key questions remain—particularly whether genome editing can increase genetic diversity without causing unintended non-target impacts. Genome editing also provokes new perspectives on conservation practices where ecosystem-wide impact assessment, case-by-case evaluations, and post-release monitoring needs to be prioritized. Furthermore, conservation biotechnology is heavily funded through private funding showing varying stakeholder interest, which can lead to untraditional and less transparent research processes. Stakeholders, including local and indigenous people, are only to a certain degree involved, which may weaken inclusion of local knowledge and monitoring efforts. Finally, concerning governance and policy, there is an urgent need to develop more adequate regulation of conservation biotechnology, as environmental release of genome-edited animals challenges definitions and guidelines in current nature protection laws and GMO regulations. Based on our analysis, we outline key points for further investigation toward a more sustainable approach to conservation biotechnology.

**Graphical Abstract:**

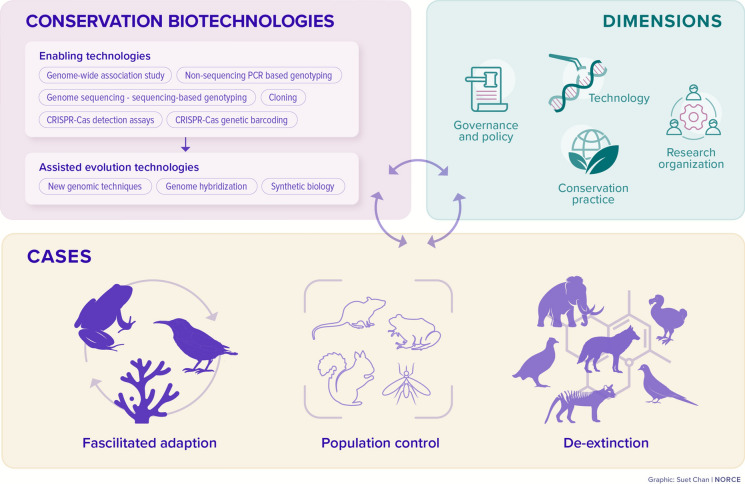

## Introduction

Human activities have since long been accelerating what has become the sixth extinction of biodiversity (Ceballos et al. 2015). Just under one million species are threatened with extinction (IPBES 2019; IUCN 2024). The planetary boundary of biodiversity has also been transgressed, in terms of both genetic diversity and functional integrity (Richardson et al. 2023). Some of the main drivers behind biodiversity decline are expanding land-use and agriculture, climate change, increased pollution, the spread of diseases and parasites, and the introduction of invasive and alien species (IPBES 2019). Efforts are being made to halt the decline, including biological conservation. The Kunming-Montreal Global Biodiversity Framework establishes goals whereby “[…] by 2050, extinction rate and risk of all species are reduced tenfold, and the abundance of native wild species is increased to healthy and resilient levels” (CBD 2022a Goal A). But current efforts are not picking up with the rapid decline in biodiversity. Phelps et al. (2020) argue that “[t]he seriousness of [the] conservation challenges requires continual development of new approaches and technologies that can be used to further conservation efforts”. This statement is supported by several others and has influenced the burgeoning of a new field of research and practice termed conservation biotechnology (Tibbetts 2022)*.* One of the many potential technologies that can be beneficial in this field are genome-editing techniques. The most widely used genome-editing tool is the CRISPR-Cas system*.* CRISPR (Clustered Regularly Interspaced Short Palindromic Repeats) refers to a segment of DNA found in bacteria and archaea that contains short repetitive sequences while Cas (CRISPR associated proteins) are enzymes that work with the CRISPR sequence to cut DNA at specific locations. This tool has mainly been used for targeted mutation in DNA with knock-out of genes or genetic sequences or for allele replacement (Hillary and Ceasar 2023). Current and proposed applications of genome editing in animals shows wide potential in both the biomedical, agri- and aquacultural sector (Blix et al. 2021; Miklau et al. 2024; van Eenennaam 2023; Wang and Doudna 2023). However, applications of genome editing in wild species are at present less explored (Kosch et al. 2022). There are three main approaches to this: (i) *facilitated adaptation* of species by improving fitness and viability in species threatened with extinction, (ii) *population control* by removal or modification of invasive or pathogenic species, or vectors of such, or (iii) *de-extinction* of lost species (Adams and Redford 2021; Harvey-Samuel et al. 2021; Novak 2018; Phelps et al. 2020).

According to a recent horizon scanning by Miklau et al. (2024), population control is at present the only application of genome editing for conservation. However, several other initiatives are ongoing (Kosch et al. 2022; Novak 2018; Phelps et al. 2020; Piaggio et al. 2017). An overview of these is crucial for understanding the potential developments in the field. In this paper, we will therefore present the emerging landscape of genome editing for facilitated adaptation, population control, and de-extinction of species. Introducing genome editing to conservation implies a new way of intervening in natural processes and ecosystems, with a novel technology and uncertainties regarding both intended and potentially unintended impacts and consequences. In addition, the use of genome editing in animals raises questions about ethical and moral justifiability (Sandler 2020; Winther et al. 2023). These concerns make it even more crucial to explore the conditions for sustainable applications. We therefore discuss the implications of using this technology, with regards to technical challenges, for conservation practices, the organization of research and funding structures. We also address the challenges posed for current GMO and nature protection legislations. Finally, based on the discussion, we give recommendations on areas that need to be further investigated to enable a responsible and sustainable approach to conservation biotechnology.

### Conservation biotechnologies

To speed up current efforts in conservation biology, Phelps et al. (2020) calls for the introduction of genome editing tools. They point out that there is a need to translate genome editing discoveries in farm animal research to ecological restoration relevance, with growing support in the literature (see e.g., Johnson et al. 2016; Phelan et al. 2021). Genome editing encompasses a range of tools and approaches that allow for targeted removal or insertion of smaller or larger genetic sequences or bases. In addition to genome editing, there are several ways of introducing new biotechnological techniques to biological conservation (Phelps et al. 2020; Phelps 2024; Johnson et al. 2016; Taylor and Gemmell 2016; Theissinger et al. 2023). In Table 1 we list some of the approaches involved in the novel field of conservation biotechnology and their respective areas of application.

**Table 1:** Biotechnology tools and innovations that can be useful in conservation practices

Technology or strategy	Objectives and possibilities
*Enabling technologies*	
Genome-wide association study and genome sequencing—sequencing-based genotyping	Monitor genetic diversity, population size, genetic markers and traits, and introgression. Using sequencing or other genomics techniques to identify organisms and/or genes that need to be introduced in population/habitat/ecosystem, using genome-wide sequencing, genome sequencing, and Amplicon Seq
Non-sequencing PCR based genotyping	Identify genetic fingerprints or DNA fragments to identify and map species occurrence and interaction networks. Next generation sequencing, eDNA sequencing, qPCR
CRISPR-Cas detection assays	Assays such as SHERLOCK (RNA-based, CRISPR-Cas13a) and DETECTR (CRISPR-Cas12a) can be used in diagnostics and detection in a variety of samples identifying nucleotide sequences
CRISPR-Cas genetic barcoding	Introduction of point mutations in genomic locations to genetically tag populations. Can also be introduced in tissue and embryos
Cloning	Use of living or preserved cells, tissues, or specimens as biological material to produce genetic identical animal by artificial methods as somatic cell nuclear transfer (SCNT), embryo cell nuclear transfer (ECNT), or by embryo splitting
*Assisted evolution technologies*	
New genomic techniques (mainly CRISPR)	Inducing somatic mutations in DNA or RNA, or germline mutations. Can be used for allele replacement, or for knock in or out function in small, short or larger sequences at targeted sites in genome, including CRISPRi, CRISPRa, base editing and prime editing
Genome hybridization	Combine (fragments of) one genome with a template genome using CRISPR-Cas. Can be applied to de-extinct parts of genomes of extinct species combined with a living relative template genome
Synthetic biology	Generate and introduce novel (natural or synthetic) traits or recreate old traits

It is important to separate the enabling technologies from the technologies which can be used to facilitate assisted evolution—active intervention in evolutionary processes (van Oppen et al. 2015). The former are non-invasive biotechnologies that can be used to monitor and identify genetic and biological biodiversity, e.g., through analysis of eDNA (Phelps 2024). This group includes both older techniques such as classical genome-wide association studies, genome sequencing and PCR, in addition to genome editing tools used for detection or genetic barcoding, as well as cloning (Kaminski et al. 2021).

The latter group available for assisted evolution are genome editing tools for introducing somatic or germline mutations in cells or tissue. This technology, often referred to as new genomic techniques, is already widely applied in agriculture, aquaculture and biomedical research (Blix et al. 2021; van Eenennaam 2023; Miklau et al. 2024; Wang and Doudna 2023). In farm animal research and breeding, CRISPR-Cas9 is the most widely applied genome editing technology compared to zinc finger nucleases (ZFN), TALEN (effector transcription factor nucleases), and meganucleases (van Eenennaam 2023). We therefore leave alternative tools and, in the continuation, focus on CRISPR-Cas.

CRISPR-Cas is a component of the immune system discovered in microbes such as bacteria and archaea and has been developed into a powerful tool for genetic engineering (Jinek et al. 2012), where the high binding ability of CRISPR enzymes can be combined with different catalytic components. Type II CRISPR effector protein Cas9 was the first developed CRISPR system and is the most widely used genome editor. In this system, a Cas9 ribonucleoprotein complex cleaves DNA (double-stranded break), guided by a single guiding RNA (sgRNA) that is designed to match the target DNA sequence through a complementary base sequence (Murugan et al. 2017). The system can bind anywhere in the genomic DNA, as long as a protospacer adjacent motif (PAM) is present. In eukaryotic cells, once the targeted site is cleaved, error-prone cellular mechanisms repair the opening. The repair mechanisms can then lead to insertions or deletions (indels), or substitutions. Mammalian cells are prone to induce non-homologous end joining (NHEJ) or microhomology-mediated end joining (MMEJ). In NHEJ, the blunt DNA ends are ligated without template, where indels can change the reading frame of the gene or remove essential sequences, causing the intended disabled (mutated) gene. Alternatively, if a template DNA strand is provided together with the CRISPR system, the cell can initiate homology-directed repair (HDR) which introduces the template sequence (e.g., a whole gene) where the double-stranded break was made. CRISPR-Cas9 can thus be used to make internal edits to the cell or create transgenic cells by introducing foreign DNA. In addition to using active Cas9, catalytically inactive Cas9 (dCas9) can be used for CRISPR Interference (CRISPRi) or CRISPR Activation (CRISPRa), which inactivates or activates a sequence respectively, but without making modifications in the genomic DNA sequence (Reviewed in Villiger et al. 2024 and Mariano Junior et al. 2024).

Beyond traditional editing, newer CRISPR-based technologies have expanded the toolkit, such as base editing and prime editing. In base editing, the structural components is a DNA-binding unit (dCas9), and a deaminase domain. Once the sgRNA binds to the target site, a single-stranded DNA loop (R-loop) is exposed, allowing the deaminase to chemically modify specific bases. Deaminated bases will mismatch with non-modified DNA, which is solved by the cell either by reverting the deaminated bases, or by sealing the new bases in the genome. The latter is stabilizing the new base in the genome and is the desired outcome for genome editing. In prime editing, the genome is edited through reverse transcription leading to small indels or nucleotide replacements. Structural components of the CRISPR system are a protein component (a Cas9 effector making single stranded breaks and a reverse transcriptase domain), and a prime editor guide RNA (pegRNA: the sgRNA scaffold and an RNA extension encoding the desired DNA edit). The pegRNA serve as a template for the reverse transcriptase. Once bound to the targeted site in genomic DNA, the Cas9 prime editors make a single-stranded break, exposing the strand which then hybridizes to the primer binding site of the pegRNA. The reverse transcriptase uses the genomic DNA as a primer, and the pegRNA as a template for synthesis. This creates a genomic flap (a 3´-flap competing with un-edited DNA) which is taken care of by the cellular repair system, ligating the new modified DNA into the genome (Reviewed by Villiger et al. 2024 and Mariano Junior et al. 2024).

In the following sections, we describe how genome editing can be applied to impact wild populations and species through facilitated adaptation, population control, and de-extinction. While other conservation biotechnology approaches exist, such as environmental detoxification (Tepper et al. 2025), this review focuses on genome-editing methods that directly alter animal populations through assisted evolution.

#### Facilitated adaptation

In conservation of threatened species, genome editing can be used to remove or introduce specific traits to improve adaptive capabilities. This approach falls under the term facilitated adaptation which aims at reinforcing a population by introducing beneficial alleles (Samuel et al. 2020), but is not limited to the use of genome editing. It encompasses a range of other conservation practices where genetics are targeted by introducing alleles, genes or individuals to a population to counteract genetic erosion, inbreeding depression, or reduced evolutionary potential as a response to the environment or threats. The strategy and trait in need of modification depends on the conservation context and specific species in question, and relevant capabilities are for example adaptability to a changing climate, emerging pathogens (Phelps 2024), or reproductive challenges in small populations. The process includes a range of enabling technologies, as well as genome editing (see Table 1).

#### Population control

Currently, the use of genome editing in conservation of wildlife is mainly focused on population control, for managing invasive species or disease vectors (Melesse Vergara et al. 2022). Different approaches are under development and research, for population suppression or modification. To skew the sex ratio of a population is one effective approach for population suppression, and can be achieved by creating all-male or all-female populations, or through spread of sterility such as sterile insect technique (Teem et al. 2020). Another method involves the use of gene drives, where a gene is spread with super-Mendelian inheritance through a population (higher than 50%). The effect depends on whether the introduced trait is reducing the fitness of the organism. In population modification, the goal is to alter specific traits without necessarily reducing population size. This can be achieved by breeding individual’s ex situ with a heritable trait and releasing them into the wild to propagate the trait (Harvey-Samuel et al. 2021). Recent advances such as Allele Sails represents a novel approach to genetic control, enabling the inheritance of a genome editor that modifies the DNA without the introduction of foreign DNA sequences (Johnson et al. 2024).

#### De-extinction

De-extinction has become known as the process in which the genome of an extant species is modified according to available genetic information of an extinct species (Novak 2018). This approach requires genetic material of the extinct species, as well as from one or more appropriate extant relative species that can be used both as a genetic template, and as a surrogate for reproduction. The process includes somatic cell genome editing or primordial genome editing in the case of avian species. The resulting offspring is a hybrid of the extinct species and the extant relative used as template. It is often termed a proxy of the extinct species as the individuals is not identical to the extinct species in terms of genetics, physiology, morphology or behavior (Novak 2018). While some refer to this process as precise hybridization, we use the term genome hybridization due to challenges associated with untargeted and non-target effects inherent in genome editing, which makes precise an inaccurate term in this context.

## Methods

To identify planned or commenced applications of genome editing in wild animals, a literature search was conducted. Two searches were performed in Google Scholar, one initial research, and one extended research. Google scholar was selected as the search engine because it provides access to both peer-reviewed scientific literature and grey literature, ensuring comprehensive coverage of available sources. The first search string was ("CRISPR") AND ("resurrect" OR "de-extinct" OR "conserve" OR "endangered" OR "rewild" OR "rewilding"), while the second was ("CRISPR" OR "genome editing" OR "gene editing" OR "precision breeding" OR "precision conservation") AND ("resurrect" OR "de-extinct" OR "conserve" OR "endangered" OR "rewild" OR "rewilding"). Searches focused on 2018–2024, as CRISPR-Cas-based genome editing only became widely used after 2012, and there is usually a delay of a few years between project initiation and public reporting. Only the first two resulting pages of records were assessed. The search was sorted according to relevance and only results with terms in title relevant to the content were included. Studies on plants and human cells were excluded, as well as papers with limited reading access. Relevant result records were then screened based on abstract, using the same exclusion criteria. Reviews were included as relevant. Literature published in 2025 has been added subsequently. Further, websites of the leading organizations within the field, Colossal Laboratories & Biosciences and Revive & Restore, were used to map relevant projects. Finally, the review results were compared to a recent horizon scanning performed by the Ad Hoc Technical Expert Group on Synthetic Biology (AHTEG) established by the Convention of Biological Diversity (CBD), which is openly available online (CBD 2024b). After this step, two more records were added to our list of projects, and one record was added on recommendation from an anonymous reviewer. The results were categorized as either facilitated adaptation, population control, or de-extinction.

## Results

### Current research on genome editing for facilitated adaptation

Conservation efforts using genome editing for facilitated adaptation are listed in Table 2. To date, the only study demonstrating proof of concept is on coral (*Acropora millepora*). Two other cases in development, the Iiwi honeycreeper (*Drepanis coccinea*) and southern corroboree frog (*Pseudophryne corroboree*), have been explored through simulations and suggested as potential strategies, and are therefore included here to illustrate potential future applications.

**Table 2:** Applications of genome editing using CRISPR-Cas for facilitated adaptation of threatened species

Species	Common name	Viability status*	Phenotype (genotype)	Country	Status	References
*Acropora millepora*	Coral	EN	E.g., heat tolerance	U.S., Australia	Proof of concept	Cleves et al. (2018, 2020), van Oppen et al. (2015), Voolstra et al. (2021)
*Drepanis coccinea*	Iiwi honeycreeper	VU	Disease resistance avian malaria	U.S	In development	Samuel et al. (2020)
*Pseudophryne corroboree*	Southern corroboree frog	CR	Disease resistance against chytridio-mycosis	Australia	In development	Kosch et al. (2019, reviewed in 2022)

^*^According to IUCN (2024) Red List. Abbreviations: EN – endangered, VU – vulnerable, CR – critically endangered

Currently, coral reef survival and development is threatened by rising temperatures and acidification in the oceans, in addition to pollution and extreme weather. These impacts are causing what is known as coral bleaching, due to the coral losing the microalgal symbiont which again causes coral death (Fisher et al. 2015; van Oppen et al. 2015; Voolstra et al 2021). Mitigation efforts are now looking at the potential for restoration through both assisted gene flow (Hagedorn et al. 2021), and targeted evolution of the coral thermal tolerance ranging from ex situ breeding and inoculation of larvae coral with stress-tolerant *Symbiodinium* microalgae*,* to mutagenesis of *Symbiodinium* symbionts. The latter approach could imply marker assisted selection of stress-tolerant strains (van Oppen 2015). More recent studies have shown the potential of CRISPR-Cas in modifying coral holobionts. Cleves et al. (2020) have used CRISPR-Cas9 for disruption of a gene regulator Heat Shock Factor 1 (*HSF1*) causing reduced heat tolerance in *A. millepora*. Similar previous experiments targeted genes coding fibroblast growth factor 1a (*FGF1*), green fluorescent protein (*GFP*) and red fluorescent protein (*RFP*) (Cleves et al. 2018). Another recent study has also demonstrated gene knock-out and genetic tagging using CRISPR-Cas in northern star coral (*Astrangia poculata)* (Warner et al. 2025). Although this latter example is not targeting conservation directly, and is therefore not included in Table 2, it shows the potential for developing genome-edited corals that are adapted to environmental pressure.

Apart from facing the direct impact of climate change, some species are indirectly affected and threatened by climate change. In Hawaii, the distribution of avian malaria by *Plasmodium relictum* vector southern house mosquito (*Culex quinquefasciatus*) threatens different forest bird species, such as the Iiwi honeycreeper. At high latitudes, malaria is limited by lower temperatures, and this is where Iiwi still survives. With a changing climate, it is expected that the temperature rises in high latitudes, potentially inducing wider distribution of the mosquito vector. Supplementing this, the Iiwi depends on nectar from flowers at lower latitudes (Samuel et al. 2020). Approaching this challenge, Samuel et al. (2020) have simulated facilitated adaptation by release of genome-edited malaria tolerant or resistant Iiwi. A malaria tolerant Iiwi would have reduced immune responses at infection and reduced malaria mortality rate as seen in the Hawaii Amakihi (*Chlorodrepanis virens*), while a resistant Iiwi would imply either enhanced immune responses or complete immunity to infection (Samuel et al. 2020). They found that the success by release of resistant Iiwi will depend on malaria transmission rates, climate projections, number of birds released and the time of release – where earlier release would be beneficial. The authors do, however, also discuss the current lack of knowledge about malaria resistance as well as other potential phenotypic effects of tackling malaria infection as tolerant or resistant.

In Australia, the southern corroboree frog is functionally extinct, with only 50 wild individuals still alive. One of the main challenges to its survival is the amphibian fungal pathogen *Batrachohcytrium dendrobatidis* causing chytridiomycosis infection*.* Kosch et al. (2019) found that the species holds genetic and phenotypic variation in susceptibility to the fungus and suggests this indicates that the frog is a good candidate for genetic engineering for disease resistance.

Other species suggested targets for genome editing are Carolina parakeet (*Conuropsis carolinensis*), Baiji river dolphin *(Lipotes vexillifer),* northern white rhinoceros (*Ceratotherium simum*), eastern quoll (*Dasyurus viverrinus*) and pink pigeon (*Nesoenas mayeri*). Additionally, some species have been targeted in conservation programs using cloning, including black-footed ferret (*Mustela nigripes*) and Przewalskiis horse (*Equus przewalskii*) (Novak et al. 2024, 2025, Revive & Restore n.d. d). There are, however, no information on planned or conducted research for genome editing these species, and they are therefore not included in the table.

### Population control

Population control is perhaps the most well-known use of genetic engineering in wild species. Here we have identified seven different species targeted for population suppression or control using genome editing (see Table 3).

**Table 3:** Applications of genome editing for population control

Species	Common name	Ecotype	Location	Phenotype	Approach	Status	References
*Culex quinquefasciatus*	Invasive mosquitos	Invasive to NZ, Hawaii and Galápagos, vector carrying avian pathogens	Globally	Sterility or eye or body pigmentation	CRISPR-Cas (amongst other) gene drive	Proof of concept	Reviewed in Harvey-Samuel et al. (2021), Harvey-Samuel et al. (2023)
*Anopheles sp.*	Mosquitos	Vector carrying Malaria after infection of *Plasmodium*	Tropical countries	Sex determination, sterilization, disease resistance	CRISPR-Cas gene drive, Ifegenia, pgSIT	Proof of concept	Adolfi et al. (2020), Smidler et al. (2023), Smidler et al. (2024), Tolosana et al. 2025
*Aedes sp.*	Mosquitos	Vector carrying arbovirus such as Dengue, Zika etc	Global	Eye or body pigmentation, disease resistance, flightless, sex determination	CRISPR-Cas, gene drive, pgSIT	Proof of concept	Anderson et al. (2024), Bui et al. (2023), Li et al. (2021), Liu et al. 2020
*Mus musculus*	House mouse	Invasive species	U.S	Single-sex-related genes	CRISPR-Cas gene drive	In development	Campbell et al. (2019), Gierus et al. (2022), Bunting et al. (2024)
*Sciurus carolinensi*	Grey squirrel	Invasive species	UK	Female fertility	CRISPR-Cas gene drive	In development	Faber et al. (2021)
*Rhinella marina*	Invasive cane toads	Invasive species	Australia	Toxicity via bufotoxin hydrolase, body pigmentation	CRISPR-Cas	Proof of concept	Cooper et al. (2020), Clark et al. (2025)
*Rhinella marina*	Invasive cane toads	Invasive species	Australia	Limited development	CRISPR-Cas	Proof of concept	(Cane Toads in Oz n.d.)
*Peromyscus leucopus*	White-footed mouse	Vector carrying ticks with Lyme disease	U.S	Disease resistance (*anti-OspA*), host compatability	CRISPR-Cas	In development	Buchthal et al. (2019), Snow (2019)

Abbreviations: Ifegenia (inherited female elimination by genetically encoded nucleases to interrupt alleles), pgSIT (precision-guided sterile insect technique

#### Population suppression

To date, mosquitoes have been widely studied as targets for genetic population control (see e.g., Harvey-Samuel et al. 2021; Tajudeen et al. 2023; Wang 2023; Weng et al. 2024). The focus has been on gene drives, but more recent applications of genome editing include approaches that do not rely on self-propagating elements. This include PgSIT (precision-guided sterile insect technique) and Ifegenia (Inherited female elimination by genetically encoded nucleases to interrupt alleles) that generate sterile males and dead females by CRISPR induced disruption of vital genes (Weng et al. 2024). Another emerging approach is Y-chromosome linked genome editors that induce male-inherited mutation generating sterility in females (Tolosana et al. 2025). Across different approaches, we identified three main groups of species targeted: the invasive southern house mosquito carrying the avian pathogens avian malaria, West Nile virus and avian poxvirus (Harvey-Samuel et al. 2023), *Anopheles sp.* which are malaria carrying mosquitos (Smidler et al. 2024; Tolosana et al. 2025)*,* and *Aedes sp*., carrying both zika virus, chikungunya and dengue fever (Bui et al. 2023). Both sterility, fertility, sex determination and pigmentation have been targeted, as well as population modification of disease resistance (Weng et al. 2024; Adolfi et al. 2020). To our knowledge, no cases involving genome editing in mosquito have yet gone to experimental field release.

Besides insect control, rodents have been targeted for genetic population suppression. The Genetic Control of Invasive Rodents partnership have suggested to use gene drives to eradicate house mouse (*Mus musculus*). According to Campbell et al. (2019) initial trials would be executed in captive settings in the U.S., Australia and New Zealand, to study the distribution of the gene drive construct in a population. More recently, Gierus et al. (2022) demonstrated proof of concept for a gene drive leading to female infertility, and thereby altering the sex ratio in a population, which would cause a decline in the population (Campbell et al. 2019). A similar study on house mouse has also been done by Bunting et al. (2024), but did not succeed in making male-biased offspring.

Faber et al. (2021) have modeled the potential for releasing gene drives to control populations of grey squirrel (*Sciurus carolinensis*). This species is invasive in the UK and especially known for being a threat to the survival of native red squirrel (*Sciurus vulgaris*). In their study, Faber et al. (2021) suggested combining three gene drives; homing, daisyfield and cleave-and-rescue, which leads to female infertility, avoids allele resistance, and ensure that the spread of the gene drive remains self-limiting for controlled release.

In Australia, the cane toad (*Rhinella marina*) is an introduced species, whose defense mechanism against predators, the lethal toxin bufagenin, is causing population declines in several predator species. Several approaches to controlling the species with genome editing is commenced. One group working under the project titled Cane Toads in Oz has developed a cane toad that is unable to metamorphose from tadpole to adulthood. As a result, these individuals are unable to reproduce and spend their whole lives as tadpoles (Cane Toads in Oz n.d.).

#### Population modification

A different approach to controlling the cane toad is to modify rather than eradicating it. Cooper et al. (2020) have shown proof of concept for reducing the ecological impact by using CRISPR-Cas knock-out of the toxin activating gene, however, this work did not achieve germline transmission. A recent study by Clark et al. (2025) provided the first demonstration of germline genome editing in cane toads. This study targeted the tyrosinase gene to enable visual identification and tracking of modified individuals across generations (Clark et al. 2025). In contrast to population suppression approaches, such as inhibiting metamorphose, the work by Clark et al. (2025) proved that it is possible to use genome editing for heritable modification that reduce the ecological impact without eliminating the species.

In the U.S., the white-footed mouse (*Peromyscus leucopus*) is functioning as a reservoir for different pathogens transmitted from deer ticks (*Ixodes scapularis*). At infection in mammals, these pathogens have complications such as Lyme disease. Buchthal et al. (2019) have as part of a larger project, Mice Against Lyme Disease, suggested that developing white-footed mice resistant to tick-borne disease caused by *Borrelia burgdorferi* could reduce local mouse and tick infection rates based on previous research on immunization through vaccination. Genome-edited Lyme disease resistant mice are planned released for facilitated adaptation at Nantucket and Martha´s Vineyard in Massachusetts as public health project aimed at reducing the spread of Lyme disease (Buchthal et al. 2019).

### De-extinction

Finally, six different projects were identified that focus on reviving species that are already lost (see Table 4).

**Table 4:** Application of genome editing using CRISPR-Cas for de-extinction of species

**Species**	**Common name**	**Original ecotype**	**Original location in situ**	**Extinct year (***before present time***)**	**Supporting species (genomes, surrogates)**	**Status**	**References**
*Thylacinus cynocephalus*	Tasmanian tiger	Apex predator, biodiversity	Australian mainland	1936	Dasyurid/fat-tailed dunnart	In development	Colossal Laboratories & Biosciences (n.d. d) Peel et al. (2021)
*Tymanuchus cupido*	Heath hen	Indicator species	Easter U.S. (Maine to Carolinas)	1932	Greater Prairie Chicken	In development	Revive & Restore (n.d. b; c)
*Ectopistes migratorius*	Passenger Pigeon	Ecosystem engineer (forest disturbance, regeneration cycle)	Eastern U.S	1914	Band-tailed pigeon	In development	Novak (2018), Revive & Restore (n.d. a)
*Raphus cucullatus*	Dodo bird	Omnivore/seed disperser	Mauritius (endemic)	1690	Nicobar Pigeon (host cell, genome template), Rodrigues Solitaire (genetics), chicken	In development	Colossal Laboratories & Biosciences (n.d. b)
*Mammuthus primigenius*	Woolly Mammoth	Ecosystem engineer (megaherbivore, grassland maintenance)	Sibiria	*3900*	African elephant, Asian elephant	In development	Revive & Restore (n.d. e), Colossal Laboratories & Biosciences (n.d. c)
*Aenocyon dires*	Dire wolf	Apex predator	U.S	*10,000*	Greywolf genome (*Canis lupus*), domestic dog surrogate	Developed	Colossal Laboratories and Biosciences (n.d. a)

The most recently extinct species targeted is the Tasmanian tiger (*Thylacinus cynocephalus*), also known as the thylacine. This marsupial apex predator endemic to Tasmania went extinct in 1936 after massive efforts to hunt it down due to it preying on sheep (Menzies et al. 2012). In the aftermath, it has become an iconic species to the island. A research group at Melbourne University is now working in collaboration with Colossal Laboratories & Biosciences to de-extinct the species. The approach involves sequencing the thylacine genome from tissue samples and using the fat-tailed dunnart (*Sminthopsis crassicaudata*) as a template and model marsupial species (Cook et al. 2021; Feigin et al. 2018). Further steps under development are to identify the genes of the dunnart which needs to be modified to match the thylacine, develop thylacine stem cells and embryo based on dunnart eggs, and transfer to appropriate surrogate (TIGRRlab n.d.).

The heath hen (*Tymanuchus cupido*) was an indicator species of the Eastern U.S. that went extinct in 1932. The template species, the greater prairie chicken (*T. c. pinnatus*), is a close relative (Palkovacs et al. 2004). The research includes developing methods for avian germline transmission and assisted reproduction (Revive & Restore n.d. c). In the same geographical area, the passenger pigeon (*Ectopistes migratorius*) was an ecosystem engineer contributing to canopy disturbance events which regenerate forest cycles. It went extinct in 1914 due to human exploitation (Nurray et al. 2017). The passenger pigeon is planned de-extinct by genome editing the necessary locations of band-tailed pigeon (*Patagioenas sp.*) germline cells which will be transferred to a developing rock pigeon (*Columba livia*), creating a chimera. After breeding the first generation, the de-extinct passenger pigeon proxy is planned to be released in the wild with implanted GPS trackers before 2040 (Revive & Restore n.d. a).

The dodo (*Raphus cucullatus*), a flightless bird endemic to the island Mauritius, went extinct in the end of the seventeenth century. Dutch settlers exploited the bird for food but also brought with them black rats (*Rattus rattus*) that ate the dodo eggs (Turvey and Cheke 2008). Colossal Laboratories & Biosciences and partners have been working on the genetic background of the Dodo and compared it to Nicobar pigeon (*Caloenas nicobarica*) and Rodriges Solitaire (*Pezophaps solitaria*) (Colossal Laboratories & Biosciences n.d. b, Soares et al. 2016). According to the Colossal webpage, this will be followed by culturing of primordial germ cells from Nicobar pigeon as a genetic template species, transfer of these cells to chicken for surrogacy, genome editing for dodo genotypic traits, before incubation and hatching (Colossal Laboratories & Biosciences n.d. b).

The woolly mammoth (*Mammuthus primigenius*), a chronospecies of the European mammoths (*Mammuthus*), went extinct approximately 3900 years BP in Arctic Siberia due to a drastically changing climate and human expansion (Lister and Sher 2001; Nogués-Bravo et al. 2008). Colossal Laboratories & Biosciences is working to create a mammoth proxy as a megafaunal ecological engineer to approach climate change mitigation (Macias-Fauria et al. 2020; Poquérusse et al. 2024). The woolly mammoth is planned revived by using Asian elephant (*Elephas maximus*) genome as a template, inserting a total of 65 identified different genes from woolly mammoth genomes necessary for the wooly mammoth phenotype. Edited Asian elephant somatic nucleus will be transferred to Asian elephant egg donor and subsequently implanted in an Asian or African elephant surrogate to create the mammoth proxy (Colossal Laboratories & Biosciences n.d. c).

The dire wolf (*Aenocyon dires*) is the most recent addition to the de-extinction projects category that has been made public. The dire wolf went extinct about 10,000 BP in North America (Perri et al. 2021). Although widely covered at the Colossal Laboratories & Biosciences home page and in news media, there are no peer reviewed publications from this project yet. According to the company, cloned pericyte blood cells of grey wolf (*Canis lupus*) were edited at 14 loci, affecting 20 different genes. Domestic dog of unknown breed was used as a surrogate, and three cubs have been shown to the public. One of the genes targeted is that of Ligand Dependent Nuclear Receptor Corepressor Like, involved in various processes such as body size and growth. The details of other genes targeted are not made publicly available, but the cubs supposedly have wider shoulders and sculls, white fur coat, and make different sounds from that of grey wolves (Colossal Biosciences & Laboratories n.d. a; Kluger 2025). The main objective for the de-extinction research on the dire wolf is the conservation of the red wolf (*Canis rufus*), and in parallel to the cloning and genome editing of grey wolf hybrids, two litters of cloned red wolf cubs have been born.

Additional projects identified in this review, but which have not yet been initiated or that lack information, include the gastric brooding frog (Yong 2013), the little bush moa (Novak 2018), and the great auk (Novak 2019). The Christmas Island rat has also been used as a model for simulation of de-extinction (Lin et al. 2022).

## Discussion

The emerging trends in genome editing of animals in the wild indicate that this technology holds potential for facilitated adaptation, population control, and de-extinction. In the following, we explore the wider implications of these developments, in relation to the technology itself, the practice of conservation, organization of the research, and frameworks of policy and governance.

### Technology

#### Genome editing advances

One of the main bottle necks for genome editing has been advancing from single-target editing to targeting multiple genes in parallel—either for targeting several traits, and/or polygenic traits where more than one gene is involved. This is often referred to as multiplex or large-scale genome editing (McCarty et al. 2020). For several of the cases described here (see Table 2–4), this will become necessary. One of these is the introduction of malaria resistance in Iwii honeycreeper (Samuel et al. 2020). Resistance to malaria is found to vary between different Hawaiian birds, and survival depends on a range of gene expressions patterns as well as the gut microbiome (Atkinson et al. 2014; Navine et al. 2023; Paxton et al. 2023; Woodworth et al. 2005). While Kosch et al. (2022) conclude that for such complex cases, selective breeding may be more efficient, others argue that multiplex genome editing in animals is feasible (Fischer and Schnieke 2023; McCarty et al. 2020; Sonstegard et al. 2024). In de-extinction, multiplex genome editing is even more crucial as several traits will differ between extinct and extant species. The mutations in the grey wolf by Colossal Laboratories & Lifesciences is a result of multiplex editing 20 different loci (Colossal Laboratories & Biosciences, n.d. a). Assumingly, most of these edits were performed in parallels within one generation, however the number of trials leading up to the successful results are not known. Furthermore, the potential for multiplex editing in de-extinction efforts might be limited by the high genetic variation between extinct species and their extant relatives.

Another advancement is base editing and prime editing (Averina et al. 2024). These methods may enhance the ability to introduce naturally occurring alleles across species, aiding facilitated adaptation. They are also effective in both dividing and non-diving as well as slowly dividing cells, such as neurons and muscle cells, enabling point mutations found in extinct species genomes, and can be used to introduce multiple edits simultaneously.

#### Genetic diversity is key

In conservation of species, diversity should be maintained as it can be an important protection mechanism for a species in overcoming future pressures. Therefore, the successful introduction of genome editing in conservation will in general depend on the potential of the technology not to reduce diversity within species. The key can also be to increase diversity, such as the case of resistance to chytridiomycosis in southern corroboree frog (Kosch et al. 2019). Using genome editing for increasing diversity might, however, run the risk of decreasing it. Like selective breeding with high-performing individuals, a focus on individual traits might reduce the genetic variation of alleles connected to other traits (Kosch et al. 2022). Kosch et al. (2022) categorize these risks as the potential loss of both targeted and non-targeted genetic variation. Inbreeding might also become a result if the targeted species needs several generations of artificial or assisted ex situ breeding after the genome editing (Doekes et al. 2021). The role of gene drives and Allele Sails could be of use here, by enabling more efficient introduction of adaptive traits, without impact on other genetic loci. Regardless of the approach, potential indirect effects are of crucial consideration and need to be acknowledged in conservation projects before introducing genome editing.

#### Unintended genetic effects

Another risk connected to the use of genome editing is potential unintended off-target and non-target effects (Aquino-Jarquin 2021; Okoli et al. 2021). Phelps (2024) has argued that off-target mutations do not matter for conservation purposes. However, depending on where these mutations occur, they may have impacts on the animal itself. This is especially relevant if the population is small, as mutations can be transferred to next generations depending on if the unintended mutation is recessive or not (Höijer et al. 2022). This points to the importance of building reference genomes (Theissinger et al. 2023), as they enable understanding of functional genetic variation of importance for fitness variation and adaptive potential, for characterization of inbreeding, and may help to reveal unintended off-target and non-target effects and if these have any detrimental impacts.

#### Cloning as tool and alternative

Cloning can be an important enabling procedure for the use of genome editing in animals. A recent review on animal cloning identified 14 initiatives of cloning for conservation purposes (Novak et al. 2025). It can also be an alternative for the introduction of genetic diversity in a population, by introducing individuals with a favorable genetic makeup to the conditions. The black-footed ferret, a North American threatened species, has been conserved through intensive conservation programs since 1985 (Novak et al. 2024). By 2019, the wild population counted 340 individuals, all of which are descendants of seven individuals (Novak et al. 2024; U.S. Fish and Wildlife Service 2019). Now, somatic cell nuclear transfer from cryopreserved cells of two individuals that have no living descendants has been performed to help increase the genetic diversity in the wild population (Novak et al. 2024). The ferret will be the first case of environmental release of cloned animals. According to some sources, see e.g., Redford and Adams (2021), genome editing of disease related genotypes are also suggested or planned for the ferret as they are vulnerable to sylvatic plague, a flea-borne bacterial disease (Novak et al. 2018). Similarly, as part of the dire wolf project, Colosssal Laboratories & Biosciences has developed an advanced cloning approach using pericyte blood cells. Besides the dire wolf project, they have cloned two litter of red wolf. This is a critically endangered species on the East Coast of the U.S., and a main objective for researching genome editing in grey wolf is the conservation of the red wolf (Colossal Laboratories & Biosciences n.d. a).

#### Transfer of knowledge across sectors

Progress of genome-edited livestock animals will provide knowledge and may drive the enthusiasm for similar approaches in conservation. Recent reviews of the species and traits currently being researched or applied show a vast range of potential applications relevant to conservation, such as disease resistance traits and climate adaptation (Miklau et al. 2024). Similarly, the IUCN (2016) suggests that technology development for de-extinction can benefit applications for conservation of extant species. This possibility for technology transfer can reflect the choices of candidate species identified here. Technology development in a few species representing different animal groups (i.e., large mammals, amphibians, avian species, insects) can provide building ground for establishing other breeding programs where genome editing is used as a tool. This potential for cross scale effects will be limited by how transferrable knowledge is between farmed and wild animals, as well as how representative closed ex situ trials are to field trials. Another challenge will be phenotypic plasticity, as environmental factors can influence the expression of the edited genes (Sundström et al. 2007).

#### De-extinction

Finally, we address some more specific issues regarding the controversial de-extinction projects. While presentations of de-extinction give the impression that lost species will be brought back as they were, the reality is that the individuals developed are hybrids or proxies of the extinct species and the relative living today, as described in Sect. 1.2.3. While the individuals are not genetically identical to the extinct species, they still might be able to fulfill some ecological functions or processes in the altered ecosystem, serving as functional proxies (Seddon et al. 2016). In this regard, gene banks and bio banks are crucial for knowledge about individual traits and species, having the function of libraries of potential edits to be made within a specific case. However, these gene and bio banks are only snapshots in time, an “evolutionary freeze”, while nature outside the bank evolves (Redford and Adams 2021). In relation to this, the main technical challenge for de-extinction is the difference to living relatives. Lin et al. (2022) used the Christmas Island rat, that went extinct between 1898–1904, as a model for studying challenges related to reconstruction of extinct species´ genomes. Their study suggests that several rounds of genome editing within a single genome may be necessary to reconstruct extinct DNA. The quality of ancient DNA, which is often very short molecules, challenges the ability to map against extant genomes (Lin et al. 2022). In addition, the de-extinction of the targeted species might also risk de-extinction of ancient pathogens (Seddon et al. 2016).

### Conservation as a practice

Genome editing of wild species, especially when used for facilitated adaptation and population control, holds great potential for being a tool that can contribute to solve some of the drivers of biodiversity decline. Although this technology is not a silver bullet, it might contribute as a crucial and radical change for conservation as a practice, with possibilities that have never been considered (Phelps et al. 2020). Potential benefits include enhancing species resilience to climate change, reducing vulnerability to emerging diseases, and controlling invasive species without resorting to culling. These applications could help maintain ecosystem stability and preserve genetic diversity in threatened populations. With such promises comes a great responsibility for ensuring sustainable use of the technology. In the following, we therefore place particular emphasis on the implications to conservation biology as a practice.

#### Framing conservation biotechnology

Since its origin, the goal of conservation biology has been to protect and enhance biodiversity (Soulé 1985). At the time when Soulé published his initial postulates of conservation biology, conservation biology was a field of research and practice where diversity mattered and was founded in systemic approaches, not on individual species and connected to charisma or rarity. Today, the functional status of the species is also considered a key concern (Barabás et al. 2022; Harvey et al. 2017). As conservation biotechnology builds on conservation biology, it is worth reflecting on what are the chosen targets for conservation. This includes questioning what a healthy ecosystem is (Skandrani 2016), whether that perspective is incorporated in prioritizations made in conservation biotechnology, and how these choices influence how conservation is perceived.

#### Changing species – changing ecosystems

The choice of candidate species matters both to the success of the specific species, but also for the wider effects on the ecosystem. Amongst the conservation cases identified in this review, the corals are chosen based on their importance to ecosystems, while the Iwii honeycreeper and the southern corroboree frog are more species-specific optimization cases. The Iwii and the frog projects have wider value for the development of methods and protocols for genome editing for disease resistance in avian and amphibian species, in addition to value for the species in question. However, optimization of one species might lead to isolation of the problem without addressing underlying ecological issues (Fischer et al. 2009). Phenotypic traits in individuals are linked to the phenotype of communities and ecosystems (Whitham et al. 2006). Thus, if the interaction between the species and ecosystem is not considered in choice of candidate species, it can be a risk both to the success of the conservation and to changes in the ecosystem. Ecosystems are in constant development and change, partly due to changing climate, and human land use. Animal species change or migrate accordingly, and species can have the ability to change their niches with migration (Nogués-Bravo 2024). This requires simulation prior to release to identify potential migration patterns, depending on species, as well as monitoring after release for identification of change in interspecies dynamic (Maschke and Gusmano 2021). Regarding de-extinction, Seddon et al. (2016) have pointed out that monitoring is needed as there are uncertainties about post-release performance and behavior, for example hybridization with extant forms and susceptibility to novel diseases. This should be a matter of concern in cases where the induced change by genome editing is heritable. Moreover, de-extinction projects create hybrid research animal models, which not only serve as proxies for extinct animals but also provide valuable platforms for advancing cloning and genome editing techniques in related, extant but threatened species.

#### Ecological function over time

The eco-evolutionary history is important to understand, not only for learning about how to avoid previous mistakes, but also for gaining understanding about the impacts by conserving or introducing a de-extinct species (Thorogood et al. 2023). Regarding de-extinction projects, Robert et al. (2017) suggests that connection in time should be guiding the choice of species, because with increasing time of being extinct, the original ecosystem changes, as well as interacting species, and perhaps even the need for the ecological function of the species in question. It is also relevant to ask whether the reason for the species being extinct has changed, or are they planned to be changed alongside the conservation initiative? If these aspects are the same and will not be changed, a species might be boosted or brought back to life, but for what price and for how long? (Redford and Adams 2021).

### Organization of a new field of research

#### Transdisciplinary collaboration

The development of conservation biotechnology as a research field requires diverse and interdisciplinary expertise. This should include experts from ecology, biotechnology, genetics, animal health, nature management, climate science, ethics, philosophy, and social sciences. In addition to integrating these fields in a broad approach, diverse stakeholders like local and indigenous communities needs to be included. Revive & Restore and Colossal Laboratories & Biosciences are two of the major drivers for applying genome editing in conservation. Several stakeholders and partners are involved by the two main actors, including funding bodies and private persons, boards and advisors, some governmental bodies, universities, zoological gardens and collections, and collaborating conservation organizations. Inclusion of stakeholders is important, however, there is a lack of transparency regarding which of these stakeholders that are representing nature and animals, and what the role these stakeholders have in the research projects as well as in management and post-release monitoring. Stakeholders can provide knowledge about local conditions and may give a more detailed understanding of the reasons behind the threats to endangered species or reasons why a species went extinct. Stakeholders are also crucial for gathering information about relevant socio-ecological systems (Stirling et al. 2018).

#### Funding sources

Funding is a crucial component of a research organization. Most of the projects identified here are either partly or fully funded by private sources. While private funding is not inherently negative, it potentially complicates the research landscape. Public funding is typically allocated through competitive processes, and requires qualitative investigation performed by experts in the field, budget reports, and outreach through open access channels. In contrast, private funding from venture capitalists, private foundations, and philanthropists may not necessarily have the same requirements as public funding. The IUCN has previously expressed concerns about the long-term stability of privately funded conservation initiatives (Seddon et al. 2016). Transparency and open access to scientific approaches, methodology, and results are necessary for learning to avoid repeated mistakes across initiatives (Gusmano et al. 2021). Such openness supports a responsible, case-by-case, and stepwise approach, regardless of whether the funding is public or private.

#### Transparency and the promises of science

Transparency is also important for identifying the main motivation and objectives of each project. In relation to what is driving applications of biotechnology in general, Heineman and Hiscox (2022) have suggested two different systems of learning cycles in biotechnology projects. In the first cycle, *technology-push*, the goal is to apply novel technology to appropriate problems. In the alternative cycle, *goal-pull*, the goal is to find a solution to a problem. Applied to conservation biotechnology, this framing might create a “promise space” where various applications of biotechnology such as genome editing are suggested as solutions. This “promise space” can also be recognized as “hope and hype” concerning novel technology (Brown 2003), or a lock-in situation where reliance lies on technological solutions (Gusmano et al. 2021). When a solution does not work out in the technology-push system, the cycle identifies new problems to apply the technology to, as opposed to identifying new solutions to the problem (Heinemann and Hiscox 2022). We believe that what system is applied in each conservation case depends on the interdisciplinarity of the individual projects, the stakeholder consortia involved, and the history of solutions sought and applied in each case. The analysis of Heinemann and Hiscox (2022) also shows that conservation biotechnology could avoid creating a “promise space” where the technology dictates the solutions, by addressing the problems first, and subsequently include a diversity of approaches together with stakeholders to identify the most sustainable and long-term solutions.

### Governance and policy

While global frameworks such as the Convention on Biodiversity (CBD) and IUCN provide guidance, governance of genome editing in conservation is not only a technical challenge but also a socio-political one. International agreements develop and set principles, but do not fully address issues connected to the implementation, which often brings local context, cultural values, and political dynamics (NASEM 2016). Effective governance must therefore move beyond international treaties and national regulation to include broader stakeholder networks, such as indigenous communities, NGOs, local governments and industry actors who shape conservation decisions in practice.

Participatory processes that build legitimacy and trust are an important part, besides regulation, in governance. Barnhill-Dilling et al. (2021) has proposed the Decision Phases Framework, which entails early stakeholder engagement in research and implementation processes. George et al. (2019) highlight the principle of Free, Prior, and Informed Consent (FPIC) for interventions affecting culturally significant species or territories, ensuring that affected communities such as indigenous people are involved before decisions are made. FPIC has been highlighted under CBD discussions on gene drives, pointing to increasing relevance in international frameworks. It is a principle rooted in human rights laws and requires ongoing dialogue and ability for communities to influence project design. Similarly, Hartley et al. (2022) has identified guiding principles for gene drive governance, including transparency, accountability, and adaptive management, while Taitingfong (2019) emphasizes the importance of indigenous knowledge systems, particularly in island ecosystems where gene drives have been proposed. Although principles has mostly been discussed with regard to gene drives, they are relevant for decisions to come in the future for other genome edited wild animals, and points also to that governance must anticipate ethical dimensions, such as animal welfare, cultural values, and unintended ecological consequences (Barnhill-Dilling and Delborne 2021; Rohwer and Marris 2018). In practical terms, this means that governance frameworks should integrate scientific risk assessment with social engagement ensuring that decision both reflect ecological and societal priorities.

In the fall of 2025, the IUCN World Conservation Congress was held in Abu Dhabi. There, members adopted a new policy on synthetic biology. The policy calls for safe and fair use of synthetic biology in conservation, with case-by-case assessment of risk and benefits to be considered independently, while also following the precautionary principle. It also highlights the importance of transparency, public communication and awareness, as well as stakeholder involvement to ensure informed decision-making and stakeholder trust (IUCN 2025).

In response to innovations in genome editing, several countries that have implemented GMO regulations and risk assessment frameworks are currently in the process of revising these. These regulations mostly cover microorganisms, plants and livestock animals (Turnbull et al. 2021). For example, in the EU, EFSA are in the process of updating the guidelines for risk assessment of animals established in 2013 (EFSA 2025), while in the U.S., guidelines covering heritable intentional genomic alteration in animals (IGAs) has been updated (FDA 2024, 2025). The goal is more efficient assessment of the use of genome editing in food and other productions (Turnbull et al. 2021), which generally involves limiting the need for notifying and/or assessing organisms and products resulting from edits where no foreign genetic material is introduced. Risk assessment procedures under national regulations handle risk to health and the environment by contained use and deliberate release to the environment, while the Cartagena Protocol under the CBD covers risks to health and the environment by transboundary movement of LMOs (living modified organisms, which includes genome-edited organisms). For several years, they have had a focus beyond agriculture and aquaculture, where gene drives have prompted debates about the adequacy of current risk assessment for long-term effects on the environment. The use of genome editing in wild animals is at present discussed under the CBD in an expert group (AHTEG) that work on horizon scanning, monitoring and assessment of synthetic biology developments (CBD 2022b, 2024a). They recommend robust risk assessment frameworks and the engagement with indigenous and local communities in cases where genome editing involves culturally or ecologically significant species. The AHTEG is continuing their work on horizon scanning, and will also do a review of risk assessments and regulatory frameworks for synthetic biology, gene drives, and genome edited animals (CBD 2025).

Despite these efforts, genome editing for wildlife conservation, excluding gene drives, remains largely unaddressed in treaties as the CBD, as well as from national and regional GMO regulation and environmental law. This regulatory gap raises critical questions about definitions of species, taxonomic classification, conservation status, and whether existing frameworks can adequately protect both genome edited wild animals and ecosystems (Allen et al. 2020; Benson 2012). Addressing these challenges will require systemic revisions to integrate environmental law, species protection, and genome-editing governance.

As previously discussed, a wide inclusion of stakeholders implies a range of different systems of funding and regulation, creating a complexity of participation. Sandler et al. (2021) emphasizes that this requires high transparency and early clarifications of expectations. Moreover, genome editing in wild animals may shape human perception of nature, potentially affecting public acceptance—an area still underexplored. In a U.S. study, Kohl et al. (2019) identified general scepticism towards genome editing in conservation, though support was slightly higher to applications improving survival of endangered species than for controlling alien species. As Rohwer and Marris (2018) argue, the ethical considerations must go beyond conservation needs to include animal welfare and moral responsibility associated with using this technology (Rohwer and Marris 2018). Adaptive assessments therefore require stakeholder and public inclusion to guide research toward more acceptable and lower risk applications. There is also a need to investigate legal and ethical aspects connected to transparency between actors and countries. This is already discussed regarding gene drives but are equally relevant for other genome edited animals that are supposed to be released to the environment. This may be a responsibility to be taken by CBD in collaboration with other initiatives that focus on conservation of biodiversity, for example the IUCN, will be essential to ensure global consistency and legitimacy in biodiversity governance.

## Conclusion

As genome editing technologies advance, they will become more efficient and targeted, potentially enhancing their application within biological conservation. The implications of introducing genome editing to conservation are wide and not limited to those topics highlighted in this article. Going forward, to ensure responsible and sustainable use, we provide some recommendations:

- Initiate simulation prior and monitoring mechanisms after release to assess the long-term impacts of genome editing on animal health, and on the genome-edited organisms´ impacts on ecosystems and biodiversity.
- Address the ethical implications of conservation biotechnology, both connected to the potential of creating new invasive species and unforeseen impacts on animal welfare.
- Ensure openness about funding sources, objectives and decision-making processes in genome-editing initiatives. Transparency builds trust and supports informed public dialogue.
- Engage with a broad range of stakeholders including indigenous people, local communities, NGOs, and the general public to discuss and elaborate on the sustainability and acceptability of genome editing.
- Establish adaptive and robust regulatory frameworks by integrating genetic, ecological, and evolutionary knowledge to ensure the safe use of genome editing in wild animals.

## Conflict of interests

The authors declare no competing interests.

## Data Availability

No datasets were generated or analysed during the current study.
